# Characterizing the Speech Breathing Kinematics of Older Adults With Presbyphonia

**DOI:** 10.1044/2026_JSLHR-26-00117

**Published:** 2026-09-15

**Authors:** Brian Saccente-Kennedy, Jessica E. Huber, Maude Desjardins, Martin Wiegand, Matthew Maddocks, Roganie Govender

**Affiliations:** a Department of Speech and Language Therapy (ENT), Royal National Ear, Nose and Throat and Eastman Dental Hospitals, University College London Hospitals NHS Foundation Trust, London, United Kingdom; b Cicely Saunders Institute of Palliative Care, Policy & Rehabilitation, King's College London, United Kingdom; c Department of Communicative Disorders and Sciences, University at Buffalo, NY; d School of Rehabilitation Sciences, Faculty of Medicine, Université Laval, Quebec City, Quebec, Canada; e Center for Interdisciplinary Research in Rehabilitation and Social Inclusion, Quebec City, Quebec, Canada; f CHU de Québec Research Center, Population Health and Optimal Health Practices Axis, Quebec City, Canada; g Department of Statistical Science, University College London, United Kingdom; h Division of Surgery & Interventional Science, University College London, United Kingdom; i Head and Neck Centre, University College London Hospital, United Kingdom

## Abstract

**Background:**

Age-related changes to laryngeal and respiratory systems contribute to presbyphonia, yet the speech breathing behaviors that accompany or exacerbate ageing voice problems remain poorly characterized. This study examined speech breathing kinematics in older adults with presbyphonia and compared them with published data from vocally healthy peers.

**Method:**

Thirty-five adults with presbyphonia (20 men, 15 women; *M*_age_ = 79.8 years) completed reading and monologue tasks in comfortable and background noise conditions while inductive plethysmography and calibrated acoustics were recorded. Primary outcome was lung volume initiation (LVI) expressed as % vital capacity (%VC) relative to end-expiratory level. Secondary measures included lung volume termination (LVT), lung volume excursion (LVE), LVE per syllable, expiratory flow, inspiratory flow, utterance length, rate of speech, and sound pressure level. Group and noise condition comparisons were made against normative cohorts using two-sample *t* tests and descriptive comparisons; mixed-effects models assessed utterance length effects.

**Results:**

Compared with typical older adults, participants with presbyphonia initiated speech at significantly lower LVI across tasks and loudness conditions (mean difference monologue: −8.8 %VC and −15.4 %VC, *p* = .004 and *p* < .001; mean difference loud monologue: −11.5 %VC, *p* = .008; mean difference oral reading: −7.8 %VC, *p* < .001). They also showed descriptively smaller LVE, shorter utterances, faster speech, higher expiratory flows, and markedly lower inspiratory flows. In noise, presbyphonic speakers increased LVI, LVE, and inspiratory flow, whereas typical older adults did not. Utterance length influenced LVI, LVT, rate of speech, and expiratory flow in both groups, though presbyphonic speakers exhibited greater expiratory flow reductions and smaller speech rate increases from already elevated baselines.

**Conclusions:**

Older adults with presbyphonia show a distinct speech breathing profile: lower LVI, reduced LVE, shorter utterances and faster rates of speech, elevated expiratory flow, and lower inspiratory flow, all of which partially normalize with increased speech intensity. These findings suggest that altered respiratory kinematics may be part of the symptomatology of presbyphonia. Clinical management should consider speech breathing alongside laryngeal-focused interventions. Future studies should include contemporaneous, well-matched controls and determine whether respiratory-focused therapies improve speech breathing patterns and voice outcomes.

The incidence of voice disorders increases with age (De Araújo Pernambuco et al., 2015; Golub et al., 2006; Pernambuco et al., 2017; Roy et al., 2007; Turley & Cohen, 2009; Wang et al., 2023). A recent meta-analysis estimated the dysphonia point prevalence for adults over 60 years of age to be 19% overall, but as high as 33% for residents in supported living and care homes (Wang et al., 2023). Older adults comprise roughly 21%–30% of the patients attending voice-specialist ear, nose, and throat (ENT) clinics worldwide (Davids et al., 2012; Takano et al., 2010), a proportion that outpaces their demographic preponderance in the general population (World Health Organization, 2015).

*Presbyphonia* refers to age-related dysphonia and is characterized by audible changes in voice quality, reduced loudness, and decreased endurance (Martins et al., 2014; Rosow & Pan, 2019). It is the most common form of dysphonia for older adults (Almutawa et al., 2024; Davids et al., 2012; Jacks et al., 2021). A diagnosis of presbyphonia is applied when voice symptoms and laryngoscopic findings are compatible with age-related change in the absence of any other structural, neurological, inflammatory, neoplastic, or systemic causes (Chang et al., 2023; Stradtman & Etter, 2022).

Evident age-related laryngeal change has long been considered a diagnostic marker of presbyphonia in the literature (Honjo & Isshiki, 1980). The features of an aged larynx, called *presbylarynx*, are well described: There is volume loss in the thyroarytenoid muscle (Mizuta, 2017; Santos et al., 2021; Sugiyama, 2017) together with thinning of the epithelium (Gonçalves et al., 2018), increased collagen deposition, and decreased elastin and hyaluronic acid (Mizuta, 2017). These changes contribute to vocal fold bowing and glottic insufficiency characterized most often as a spindle-shaped glottal gap (Sugiyama, 2017). However, many older adults exhibit presbylaryngeal features without developing symptomatic presbyphonia. For example, Crawley and colleagues identified age-related laryngeal change in approximately 80% of both presbyphonic individuals and normophonic controls (Crawley et al., 2018). This weak association between structure and symptom suggests that laryngeal aging alone cannot fully explain the emergence or severity of presbyphonia and that other physiological subsystems must be considered within a diagnostic framework.

Voice production is a multisystem behavior requiring the coordinated interaction and neural control of laryngeal, respiratory, and resonance/articulatory systems. As the respiratory system provides the power source for voice production, age-related respiratory decline may contribute significantly to the pathophysiology of presbyphonia. Generating and regulating subglottal pressure depends on lung elasticity, chest wall mechanics, and inspiratory and expiratory muscle strength—systems known to decline with age. The lung parenchyma becomes progressively more distensible and less elastic (Frank et al., 1957; Janssens et al., 1999; Niewoehner et al., 1975), the chest wall becomes less compliant due to calcification of costal cartilages and increased kyphosis (Mittman et al., 1965), the thorax becomes more barrel shaped (Janssens et al., 1999), and sarcopenia contributes to a reduction of size and number of respiratory muscle fibers, leading to decreased maximal inspiratory and expiratory pressures (Enright et al., 1994). These structural changes result in functional declines including increased residual volume (the volume in the lungs after maximal expiratory effort), decreased vital capacity (VC; the maximum volume that can be expelled from the lungs following a maximal inhalation), and elevated functional residual capacity (also called end expiratory level [EEL], the volume left in the lungs after passive tidal expiration; Janssens et al., 1999). Collectively, these changes alter both the pressure–volume relationships that underpin subglottal pressure generation and the available lung volume for speech. Crucially, these physiological changes must be expressed behaviorally during speech, requiring speakers to adapt how they initiate, sustain, and terminate phonation.

*Speech breathing* represents this functional link between respiratory physiology and voice production: It is the intentional and adaptive use of the respiratory system to generate and regulate subglottal pressure during speech. In healthy aging, speech breathing is characterized by compensatory adjustments to age-related respiratory changes. Older speakers initiate and terminate speech at higher lung volumes than younger speakers to optimize reduced passive recoil pressures (Hoit et al., 1989; Hoit & Hixon, 1987; Huber, 2008; Huber & Spruill, 2008; Sperry & Klich, 1992). This strategy provides greater usable volume before reaching mid lung volumes (just above EEL), where recoil pressures become insufficient and must be supplemented by more effortful expiratory muscle activation (Huber & Darling-White, 2017). Older adults also expend a greater proportion of lung volume per utterance, likely reflecting reduced laryngeal valving associated with vocal fold bowing (Hoit et al., 1989; Hoit & Hixon, 1987); this inefficiency is partly compensated by producing shorter utterances (Hoit & Hixon, 1987; Huber & Spruill, 2008). Both younger and older adults adjust their respiratory patterns in anticipation of linguistic demands (e.g., increasing lung volume initiation [LVI] for longer utterances), reflecting neural planning processes (Huber, 2008; Winkworth et al., 1994, 1995), although older speakers may exhibit reduced flexibility under increased cognitive or loudness demands (Huber & Spruill, 2008). Taken together, these findings show that speech breathing is not a fixed output of physiology, but an adaptive behavior shaped by physiological constraints.

Despite the clear relevance of respiratory function and speech breathing adaptations to voice production in older adults, the respiratory contributions to presbyphonia remain understudied. Our systematic review of presbyphonia voice therapy found that respiratory outcomes were reported in only 17% of studies (Saccente-Kennedy et al., 2024). Emerging evidence suggests that this omission is consequential: Vaca et al. (2015) observed that respiratory impairment (VC or peak expiratory flow < 80% predicted values) was the most common presentation in older patients with ageing voice deficits and that respiratory impairment alongside a glottic gap produced the shortest maximum phonation times, the worst voice quality, and the highest self-reported handicap. Similarly, Desjardins and colleagues found that nearly one third of adults with presbyphonia had impaired spirometry (forced VC and forced expired volume in 1 s < 80% predicted values) and ~19% had low maximal expiratory pressures, both of which appeared to compound the effects of vocal fold atrophy (Desjardins et al., 2022a; Desjardins et al., 2022c). These findings suggest that respiratory impairment may be prevalent in presbyphonia and may exacerbate vocal deficits; however, the mechanism through which this occurs remains unclear. In particular, spirometry and respiratory muscle strength reflect maximal physiological capacity, whereas speech production operates within a submaximal functional range (Hixon et al., 1973, 1976).

A key conceptual distinction is therefore between physiological capacity (what the respiratory system can do) and behavioral adaptation (how it is used during speech). Although existing studies suggest that respiratory impairment may be common in presbyphonia, how speakers deploy their available respiratory resources during speech and whether these patterns differ from typical aging remain unclear.

Altered speech breathing patterns have been documented in individuals with respiratory and neurological conditions. For example, people with asthma use a larger fraction of their (reduced) VC, with slower inspiration and faster expiration, thought to indicate a prioritization of gas exchange over communicative efficiency (Loudon et al., 1988). In chronic obstructive pulmonary disease (COPD), speech is produced on prolonged, low-flow expirations with low lung volume terminations (Binazzi et al., 2014). In Parkinson's disease, as the disease progresses, there is a shift toward initiating and terminating utterances at lower lung volumes than controls, requiring greater expiratory muscle pressure and increasing speech effort and fatigue (Huber & Darling, 2011; Huber & Darling-White, 2017). Dysarthric speakers post–head injury demonstrate paradoxical chest wall movements during speech consistent with discoordination of rib cage (RC) and abdomen (AB; Murdoch et al., 1993).

Altered speech breathing patterns have also been identified in dysphonic speakers who are otherwise healthy. Women and children with bilateral nodules and other phonotraumatic lesions initiate speech at higher lung volumes and use larger lung volume excursions (LVEs) than controls, apparently to compensate for inefficient laryngeal valving (Sapienza & Stathopoulos, 1994; Schaeffer et al., 2002). Conversely, speakers with primary muscle tension dysphonia (pMTD) often speak at relatively low lung volumes with shortened breath groups and increased perceived effort; training them to initiate and terminate utterances at higher lung volumes improves acoustic and perceptual voice measures and reduces effort, implying that their baseline patterns were maladaptive rather than compensatory (Lowell et al., 2008, 2025).

Taken together, these findings support the view that altered respiratory kinematics can serve either as adaptive strategies to maintain phonation under physiological constraint or as inefficient, effort-increasing maladaptive patterns, and that this behavioral layer may help explain heterogeneity in symptom severity and presentation over and above underlying physiological capacity. Identifying speech breathing patterns in presbyphonia is, therefore, crucial both for understanding variability in symptom presentation and for informing targeted interventions.

Addressing this gap requires direct characterization of how respiratory resources are deployed during functional speech, rather than inferring behavior from maximal physiological measures. We therefore aim to (a) compare LVI, our primary outcome, and related metrics (lung volume termination [LVT], LVE, LVE per syllable [LVE_SYL_], utterance length, rate of speech, inspiratory flow, and expiratory flow) between presbyphonic participants and published normative cohorts, and (b) examine the effects of utterance length and loudness condition on these speech breathing metrics to determine how increased task demands influence respiratory strategies. We hypothesize that individuals with presbyphonia will exhibit age-atypical speech breathing kinematics and that these differences will be amplified under increased task demands, although no directional predictions are made in the absence of prior data in this population.

## Method

### Participants

The present study was approved by the NHS Health Research Authority (West London & GTAC Research Ethics Committee, Ref: 24/PR/0948). Participants were older adults (≥ 50 years of age) with an ENT surgeon-confirmed diagnosis of presbyphonia. They were identified from the laryngology and voice clinics of five London hospitals (Royal National ENT and Eastman Dental Hospitals, Charing Cross Hospital, Northwick Park Hospital, University Hospital Lewisham, and Whittington Hospital). Informed, written consent was obtained from all participants in advance of screening against eligibility criteria. Following successful screening, all participants underwent comprehensive voice, laryngeal, and respiratory assessment, including perceptual ratings, acoustic measurements, aerodynamic assessment, spirometry and respiratory muscle strength assessment, laryngeal imaging, and self-reported measurement of vocal handicap. Full details of these procedures are provided in the subsequent Characterization Measures subsection*.*

Inclusion criteria were being 50 years of age or older, having laryngeal exam images available for analysis, passing a hearing screen (40 dB at 500, 1000, and 1500 Hz, with hearing aids if worn), and demonstrating no significant cognitive impairment (scoring > 22 on the Rowland Universal Dementia Assessment Scale; Storey et al., 2004). Exclusion criteria were the presence of additional laryngeal pathology unrelated to presbylarynx, having a diagnosis of a neurological condition that might affect either laryngeal or respiratory function, and being contraindicated for spirometry.

Respiratory comorbidities (e.g., asthma, COPD) were not used as exclusion criteria. This decision reflects evidence that respiratory impairment has been identified to be common in presbyphonia and may contribute to symptom severity. Excluding these individuals would have reduced the ecological validity of the sample and obscured the real-world variability clinicians encounter. We acknowledge that respiratory disease may influence speech breathing metrics, and we address this potential confound in the Discussion section.

### Characterization Measures

All participants were asked about their smoking history and their respiratory comorbidities and underwent a standardized multidimensional voice and respiratory assessment protocol to document the severity and characteristics of their dysphonia (refer to the Instrumentation section for details regarding instrumentation). The measures collected are detailed below and summarized in Table 1.

- **Voice-related quality of life:** Participants first completed the Aging Voice Index (Etter et al., 2019), a validated measure of voice-related quality of life in older adults (score range: 0–92, with higher scores reflecting poorer self-reported voice outcomes). The mean score for participants (38.8) indicates a moderately reduced voice-related quality of life and closely matches the mean score for people with presbylarynx (37.8) reported in the tool's validation study (Etter et al., 2019).
- **Acoustic measures:** The mean Acoustic Voice Quality Index (AVQI; Barsties v. Latoszek et al., 2021) was calculated from three tokens of sustained /a:/ and continuous speech (the first 22 syllables of The Rainbow Passage) using VoxPlot Version 10.0.26100 (https://voxplot.lingphon.com). AVQI is expressed as a value between 0 and 10 (higher values represent a more severely discrepant voice quality), with the English language threshold between nondysphonic and dysphonic voices being 1.17 (Castillo-Allendes et al., 2023). The AVQI for 83% (*n* = 29) of participants was above this normative threshold, and the mean value for the cohort was 2.79.
- **Perceptual evaluation:** The same three sustained-vowel and connected-speech tokens were rated for Consensus Auditory-Perceptual Evaluation of Voice overall severity (Zraick et al., 2011) by five trained voice specialist speech-language pathologist raters averaging 18 years of experience (range: 5–31). Raters were blinded to participant age and comorbidities, but aware of their diagnosis of presbyphonia. Seven recordings (20%) were duplicated to assess intrarater reliability. Using two-way mixed-effects, single-measure, absolute-agreement intraclass correlation coefficients (ICCs), interrater reliability was excellent (.891), and intrarater reliability was also excellent (.815–.998; *M* = 0.916). The mean overall severity for the cohort was 36.3, indicating a moderately discrepant perceptual voice quality.
- **Aerodynamic measures:** Mean estimated subglottic pressure, mean airflow during voicing, and mean laryngeal resistance were determined from three tokens of voiceless labial stop syllable trains (/pa/). Standardized *z* scores were produced with reference to age- and sex-based norms (Zraick et al., 2012) to assist interpretation. *z* scores for the participants indicated that, overall, mean airflow and median aerodynamic resistance values for the cohort were close to norms but the mean subglottic pressure was comparatively elevated (*z* score: 1.33).
- **Spirometry and respiratory muscle strength:** Quality-assured spirometry followed the Association for Respiratory Technology & Physiology guidelines (Sylvester et al., 2020) to obtain forced vital capacity (FVC), forced expiratory volume in 1 s (FEV₁), and the FEV₁/FVC ratio. *z* scores were calculated using Global Lung Function Initiative 2012 reference equations to identify values below the lower limit of normal (LLN), identified as the 5th percentile, or *z* ≤ −1.64 (Quanjer et al., 2012). Volitional respiratory muscle strength testing followed the American Thoracic and European Respiratory Societies standards (American Thoracic Society/European Respiratory Society, 2002), with maximum inspiratory and expiratory pressures (MIPs and MEPs) recorded in cmH₂O and compared with reference equations for older adults (Enright et al., 1994) to identify respiratory muscle strength below LLN. While raw spirometry values for the cohort demonstrated expected sex differences, 20% of participants were identified to have obstructive spirometry with FEV₁/FVC below −1.64. No participant had an MIP below LLN, but 23% had an MEP below LLN, being more common in males (35%) than females (6.7%).
- **Laryngeal imaging:** Videostroboscopy exams were reviewed to document vocal fold bowing, glottic closure, and supraglottic activity. Fiji software (Schindelin et al., 2012) was used by B.S.K. to calculate the total bowing index (BI), a combined measure of the concavity of both vocal folds (Omori et al., 1997), and the normalized glottic gap area (NGGA), a measure of the glottal gap at maximum closure during sustained /i:/ (Omori et al., 1997). The BI and NGGA for a random sample of 20% of participants (seven) were recalculated approximately 1 month after the original analysis with no reference to earlier determinations. Intrarater reliability was again assessed utilizing ICC and was found to demonstrate moderate (BI: 0.732) to excellent (NGGA: 0.966) consistency. Medial and anteroposterior supraglottic compression was rated on a 0–5 scale using Voice-Vibratory Assessment With Laryngeal Imaging (VALI; Poburka et al., 2017) by four experienced raters in a consensus, group rating process. A random 20% of images (seven) were rerated to assess reliability. Weighted Cohen's kappa indicated moderate agreement across repeated ratings: κ = .650 for anteroposterior and κ = .512 for mediolateral compression. Mean BI scores for the cohort indicated similar vocal fold bowing to two other studies of older adults with presbyphonia (Desjardins et al., 2022a; Kaneko et al., 2015), while median NGGA values indicated that despite this, 57% (*n* = 16) of participants had full glottic closure. VALI scores indicated that 97% of participants had some degree of supraglottic hyperfunction with median scores indicating this was mild–moderate on average.

**Table 1.** Participant characteristics: mean (95% CI)/median (95% BCa CI).

Characteristics	Presbyphonia			Control groups	
	All (*N* = 35)	Males (*n* = 20)	Females (*n* = 15)	Hoit (*n* = 20)	Huber (*n* = 23)
*M*_age_ (*SD*; range)	80 (9.5; 63–101)	81 (9.9; 63–101)	78 (9.0; 65–91)	(72–78)	71.7 (4.4; 66–82)
Ethnicity (*N*)					
Caucasian	94% (33)	95% (19)	93% (14)	100% (20)	—
Black African	2.9% (1)	0.0% (0)	6.7% (1)	0.0% (0)	—
Asian Pakistani	2.9% (1)	5.0% (1)	0.0% (0)	0.0% (0)	—
Smoking history (*N*)					
Current	3% (1)	0% (0)	7% (1)	0% (0)	0% (0)
Past	57% (20)	55% (11)	60% (9)	—	—
None	40% (14)	45% (9)	33% (5)	—	—
Median pack years	8.0 [4.0, 13.0]	12.5 [12.5, 12.5]	6.0 [1.5, 26.0]	—	—
Mean years quit	36 (26, 46)	31 (15, 47)	43 (29, 56)	—	—
Respiratory diagnoses (*N*):					
Asthma	20% (7)	20% (4)	20% (3)	0.0% (0)	0.0% (0)
COPD	11% (4)	10% (2)	13% (2)	0.0% (0)	0.0% (0)
Bronchitis	2.9% (1)	5.0% (1)	0.0% (0)	0.0% (0)	0.0% (0)
Pneumonia	0.0% (0)	0.0% (0)	0.0% (0)	0.0% (0)	0.0% (0)
Bronchiectasis	5.7% (2)	5.0% (1)	6.7% (1)	0.0% (0)	0.0% (0)
Lung cancer	0.0% (0)	0.0% (0)	0.0% (0)	0.0% (0)	0.0% (0)
Interstitial disease	2.9% (1)	5.0% (1)	0.0% (0)	0.0% (0)	0.0% (0)
Participant-reported measures					
AVI (0–98)	38.8 [33.0, 44.6]	36.8 [28.2, 45.4]	41.5 [33.1, 49.8]	—	—
Voice quality					
AVQI (0–10):	2.79 [2.23, 3.35]	3.00 [2.10, 3.91]	2.50 [1.88, 3.13]	—	—
ULN	29/35 (83%)	16/20 (80%)	13/15 (87%)	—	—
Overall severity (0–100):	36.3 [30.3, 42.4]	35.0 [26.6, 43.5]	38.1 [28.4, 47.8]	—	—
Laryngeal aerodynamics					
Airflow:					
L/s	0.17 [0.12, 0.21]	0.20 [0.13, 0.27]	0.12 [0.08, 0.17]	—	—
*z* score	−0.05 [−0.34, 0.25]	0.01 [−0.30, 0.32]	−0.12 [−0.73, 0.50]	—	—
Subglottic					
cmH_2_O	10.2 [9.1, 11.2]	10.8 [9.4, 12.2]	9.3 [7.7, 10.9]	—	—
Pressure:					
*z* score	1.33 [0.81, 1.85]	2.09 [1.37, 2.82]	0.32 [−0.05, 0.68]	—	—
Resistance:					
cmH_2_O/(L/s)	83 [53, 200]	55 [32, 133]	86 [72, 247]	—	—
*z* score	0.38 [−0.25, 3.41]	0.64 [−1.15, 6.97]	0.12 [−0.16, 3.21]	—	—
Laryngeal morphology	(*n* = 33, NGGA *n* = 28)	(*n* = 19, NGGA *n* = 15)	(*n* = 14, NGGA *n* = 13)	—	—
BI	7.94 [6.26, 9.61]	8.33 [5.90, 10.8]	7.40 [4.87, 9.93]	—	—
NGGA	0.00 [0.00, 0.13]	0.00 [0.00, 0.26]	0.00 [0.00, 0.45]	—	—
VALI_AP_	1.91 [1.56, 2.26]	2.21 [1.74, 2.68]	1.50 [1.01, 1.99]		
VALI_MED_	2.18 [1.76, 2.60]	2.58 [1.97, 3.19]	1.64 [1.16, 2.13]		
Spirometry					
FVC:					
Liters	2.91 [2.60, 3.22]	3.41 [3.02, 3.81]	2.23 [2.01, 2.45]	—	—
*z* score	−0.20 [−0.52, 0.11]	−0.11 [−0.63, 0.41]	−0.32 [−0.65, 0.00]	—	—
LLN	2/35 (5.7%)	2/20 (10%)	0/15 (0%)	0/20 (0%)	0/23 (0%)
FEV_1_:					
Liters	2.02 [1.80, 2.25]	2.32 [1.98, 2.66]	1.63 [1.50, 1.76]	—	—
*z* score	−0.58 [−0.93, −0.24]	−0.61 [−1.19, −0.02]	−0.55 [−0.85, −0.25]	—	—
LLN	4/35 (11%)	4/20 (20%)	0/15 (0%)	0/20 (0%)	0/23 (0%)
FEV_1_/FVC ratio:	0.69 [0.66, 0.72]	0.67 [0.62, 0.72]	0.71 [0.67, 0.76]	—	—
*z* score	−0.79 [−1.16, −0.42]	−0.88 [−1.44, −0.32]	−0.67 [−1.16, −0.18]	—	—
LLN	7/35 (20%)	4/20 (20%)	3/15 (20%)	0/20 (0%)	0/23 (0%)
ULN	0/35 (0%)	0/20 (0%)	0/15 (0%)	—	—
MEP:					
cmH_2_O	79.2 [70.9, 87.5]	84.6 [75.1, 94.2]	72.0 [56.7, 87.2]	—	—
LLN	8/35 (23%)	7/20 (35%)	1/15 (6.7%)	—	—
MIP:					
cmH_2_O	58.8 [50.8, 66.8]	65.5 [54.8, 76.1]	49.9 [37.8, 62.0]	—	—
LLN	0/35 (0%)	0/20 (0%)	0/15 (0%)	—	—

*Note.* Dashes indicate data not reported. CI = confidence interval; BCa CI = bias-corrected and accelerated bootstrap confidence intervals; COPD = chronic obstructive pulmonary disease; AVI = Aging Voice Index; AVQI = Acoustic Voice Quality Index; ULN = above the upper limit of normal; BI = bowing index; NGGA = normalized glottic gap area; VALI = Voice-Vibratory Assessment With Laryngeal Imaging; VALIAP = VALI anterior–posterior compression rating; VALIMED = VALI medial compression rating; FVC = forced vital capacity; LLN = below the lower limit of normal; FEV_1_ = forced expiratory volume in 1 s; MEP = maximum expiratory pressure; MIP = maximum inspiratory pressure.

### Speech Tasks

Two speech tasks were elicited from participants, reading aloud *The California Passage* (Hoit & Hixon, 1987), containing 12 sentences ranging from seven to 53 syllables, and a 90-s extemporaneous monologue on a topic of the speaker's choosing. This sequence of tasks were carried out first at comfortable loudness and then in background noise to elicit louder speech via the Lombard effect (Lane & Tranel, 1971). Leveraging this automatic, psychoacoustic phenomenon has been found to produce a larger increment in speech loudness in older people than direct cues to be louder (Sadagopan & Huber, 2007). For the background noise condition, a multispeaker babble noise (AUDiTEC, St. Louis) was played through calibrated free-field speakers placed 100 cm to the left and right of the participant at 70 dBA, with participants given no other explicit instructions than to ensure they could be understood by the researcher standing approximately 2 m away.

### Instrumentation

All data were acquired in a large sound-treated room at the Royal National ENT and Eastman Dental Hospitals, London. Hearing screening was conducted using calibrated pure tones presented at 40 dB HL at 500, 1000, and 1500 Hz in the free field via a GN Otometrics Astera Clinical Audiometer (Natus). Acoustic signals were recorded using a head-mounted DPA 6066 omnidirectional microphone, offering a flat frequency response from 20 Hz to 20 kHz and acquired via a Computerized Speech Lab Model 4500 (KayPENTAX) at a 44.1-kHz sampling rate. For accurate reporting of sound pressure level (SPL), the microphone was calibrated as per Švec and Granqvist (2018) with a Model 4230 calibrator (Bruel & Kjær), which produces a 1-kHz, 94-dB calibration tone. Laryngeal aerodynamics were obtained via the Voicing Efficiency protocol of a Pentax Medical Phonatory Aerodynamic System, PAS 6600 (Pentax Medical). Spirometry and respiratory muscle strength assessment were carried out using a Pneumotrac with RMS (Respiratory Muscle Strength) Spirometer (Vitalograph). Experimental kinematics data were acquired at a sampling rate of 22 kHz per channel using a National Instruments USB 60003 DAQ via a custom MATLAB application built by the first author within the MATLAB 2024b runtime environment (MathWorks). Respiratory kinematic data were transduced with an Inductotrace inductive plethysmograph (Ambulatory Monitoring, Inc.) on its direct current–coupled setting. The analogue voltage output of a pneumotachograph-based spirometer (Model SPR-BTA, Vernier) was used in the respiratory kinematic calibration procedures detailed below.

### Speech and Respiratory Measurements

All measurements and data collection were carried out by the first author, B.S.K. For speech breathing kinematics assessment, elasticated transducer bands were placed around the participant's RC (just under the axilla) and AB (just under the last rib and at the level of the umbilicus), with a retaining body stocking worn on top of these to keep them in place. The participant remained in a seated position throughout the assessment and were regularly reminded to keep arm movement and trunk position adjustments to a minimum, as these can impact the data acquired. The voltages produced by the transducer bands correspond to changes in their cross-sectional diameter as the participant breathes in and out.

The respiratory apparatus can be modeled as a system consisting of two degrees of freedom, with the RC and AB compartments contributing separately to lung volume displacements in breathing and speech (Konno & Mead, 1967). To convert meaningfully from the sum of the voltages recorded for each transducer band to a physiological measurement, such as liters, a calibration must be undertaken for each participant. Although several methods exist for determining such a calibration, the *least squares method* (Chadha et al., 1982), found to produce the smallest calibration errors in older adults (McKenna & Huber, 2019), was used to solve for the correction factors *k*_1_ and *k*_2_ by the following equation:

$${\text{Volume}}_{\text{spirometer}}=\left(k_{1}\times \mathrm{RC}\right)+\left(k_{2}\times \mathrm{AB}\right)$$ (1)

For this, 60-s samples of tidal rest breathing and “speech like breathing” were collected from participants concurrently with airflow transduced from a spirometer (see McKenna & Huber, 2019, and Huber et al., 2005, for details). The MATLAB *lsqnonneg* function was used to solve for a nonnegative linear least-squares regression (using the Lawson–Hanson fitting method) between the summed RC and AB signals and the actual lung volumes integrated from the airflow signal, solving for *k*_1_ and *k*_2_. A nonnegative solution was sought as, while it may be mathematically possible that best fit regression might produce a negative correction factor (*k*) for either RC or AB, it is physiologically impossible that a positive increase in either compartment's cross-sectional diameter would represent a decrease in the volume it contributes to VC.

Drifting of the integrated spirometer volume signal, caused by the expansion of ambient room air when it enters the lungs, was corrected by applying the body temperature, saturated pressure factor to inspired air volumes via the following:

$${\text{Volume}}_{\text{BTPS}}={\text{Volume}}_{\text{Ambient}}\times \frac{P_{B}-P_{\text{Room}\left(H_{2}O\right)}}{P_{B}-P_{\text{Body}\left(H_{2}O\right)}}\times \frac{273+T_{\text{Body}}}{273+T_{\text{Room}}}$$ (2)

where *P_B_* is barometric pressure, *P*_H2O_ is the water vapor partial pressure at room or body temperature, and *T* is room or body temperature in Celsius. Body temperature was assumed to be 37°C, and room temperature and barometric pressure were identified from an in-office weather monitor (Youshiko Weather Station Model YC9331, Youshiko Ltd).

Once RC and AB correction factors had been determined, participants were asked to perform three trials of a VC maneuver (maximal inhalation and exhalation) from which plethysmography-estimated VC was determined. LVIs and LVTs for utterances were then determined from the onset and termination of speech identified from the acoustic signal. This was done semi-automatically using MATLAB's *detectSpeech* function followed by manual correction of any inaccuracies as required. LVIs and LVTs were calculated with reference to EEL (identified from a minimum of three steady tidal breath minima prior to the start of each speech task) and expressed as a fraction of total VC (%VC). Volume inhaled (in %VC) was determined as the difference between the nearest volume peak preceding LVI (if not the point of LVI) and the nearest volume trough preceding this peak (see Figure 1). The acoustic signal was also used to determine SPL and to provide an English transcription using MATLAB's *speech2text* function with the *wave2vec 2.0* pretrained speech client model. To determine utterance length in syllables, the transcript was automatically segmented into syllables by comparison with a publicly available list of 62,000 syllabized English words (sources: Darby, 2018; Ward, 2002) followed by manual correction as required. The following derived metrics were then calculated:

$$\text{Lung volume excursion},\mathrm{LVE}\;\left(\%\mathrm{VC}\right)=\mathrm{LVI}-\mathrm{LVT}$$ (3)

$$\text{Lung volume excursion}\;\mathrm{per}\;\text{syllable},{\mathrm{LVE}}_{\mathrm{SYL}}\left(\%\mathrm{VC}/\mathrm{syl}\right)=\mathrm{LVE}/\text{utterance length}$$ (4)

$$\text{Expiratory flow}\;\left(\%\mathrm{VC}/s\right)=\mathrm{LVE}/\text{utterance duration}$$ (5)

$$\text{Inspiratory flow}\;\left(\%\mathrm{VC}/s\right)=\text{volume inhaled}/\text{inhalation duration}$$ (6)

$$\text{Rate of speech}\;\left(\mathrm{syl}/s\right)=\text{utterance length}/\text{utterance duration}$$ (7)

**Figure 1.**
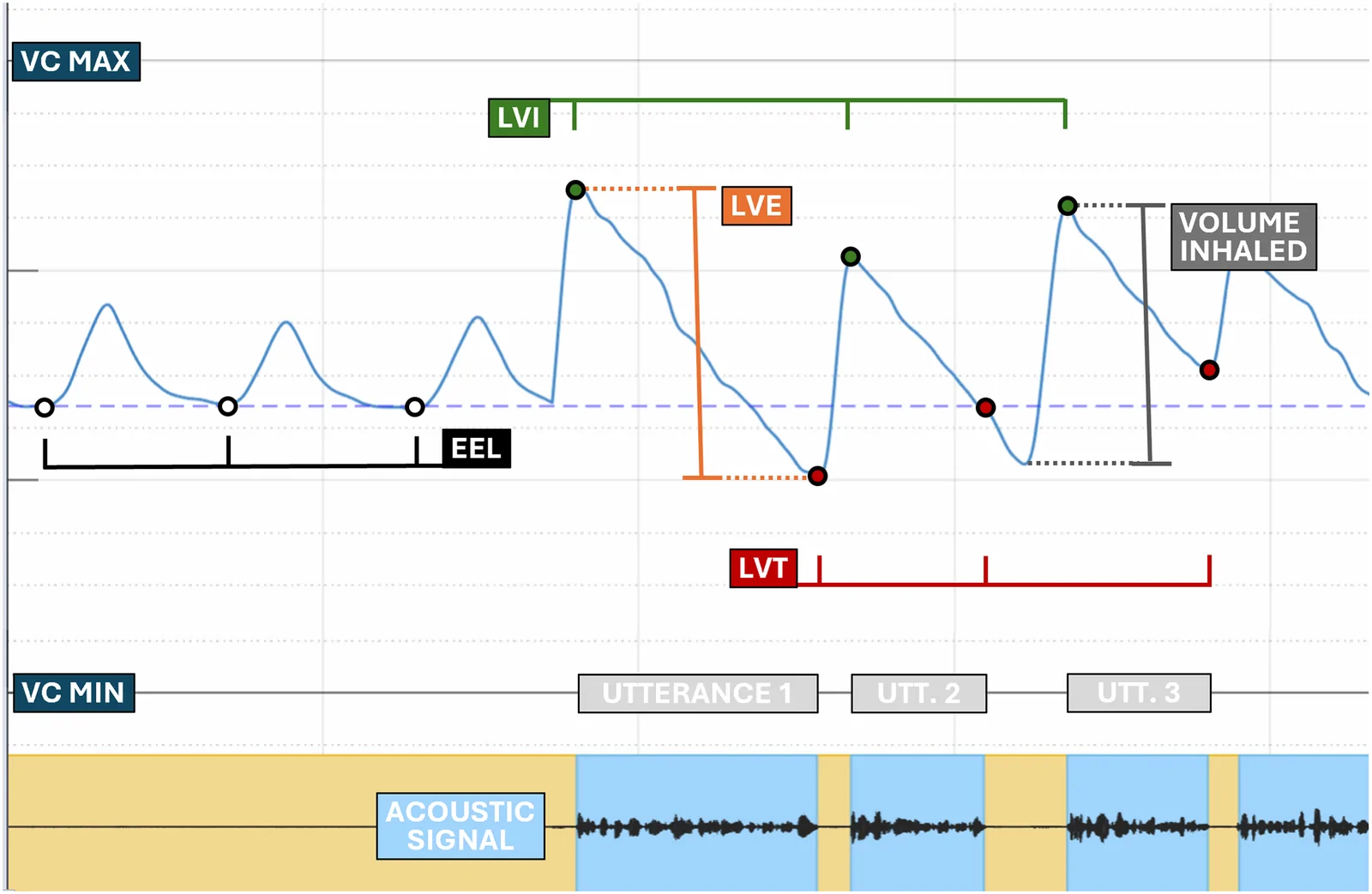
Lung volume metrics (end expiratory level [EEL], lung volume initiation [LVI], lung volume termination [LVT], lung volume excursion [LVE], inhaled volume). MAX = maximum; UTT = utterance; MIN = minimum.

### Comparison Data Sets

To contextualize the speech breathing patterns observed in older adults with presbyphonia, we compared the experimental data to previously published normative data sets. These two historical data sets served complementary analytic purposes: One provides well-established group-level normative benchmarks for speech breathing in older adults, while the other additionally affords utterance-by-utterance, individual-level analysis across different loudness conditions. A single prospectively collected control group would not have permitted both types of comparison without substantially increasing sample size and resource burden.

Comparator data arise from three published sources; Hoit and Hixon (1987), source of normative monologue and reading data for older adult males; Hoit et al. (1989), which reported normative monologue and reading data for older adult females; and Huber (2008), which provided normative monologue data collected under both comfortable and background noise conditions for older adult males and females. Available demographic information for these control participants (as reported in the original publications) is summarized in Table 1.

For the first two sources, hereafter referred to as the Hoit data sets, we combined group-level aggregate data (means and estimated 95% confidence intervals [CIs]) for the 75-year-old male (*n* = 10) and female (*n* = 10) cohorts. This approach was justified because the original authors demonstrated that speech breathing measures did not differ by sex once normalized to VC (Hoit et al., 1989). For the Hoit data sets, both monologue and reading speech tasks were elicited only in a comfortable loudness condition, and data were only available for LVI, LVT, LVE, LVE_SYL_, and utterance length. As no loud speech condition was included in the Hoit data sets, loudness comparisons for the oral reading task were restricted to within-group contrasts for the presbyphonia cohort.

In contrast, the data set reported by Huber (2008) provided breath-by-breath, individual-level kinematic data collected using the same respiratory instrumentation and calibration procedures employed in the present study. These data were collected between 2003 and 2005 at the Purdue University Motor Speech Laboratory from 23 older adults (14 females participants). Speech tasks involved extemporaneous monologue produced under both comfortable and background noise conditions, allowing extraction of the full suite of derived respiratory metrics and enabling utterance-level modeling of loudness and length effects.

Control participants across all three data sets were reported to have normal speech, language, and perceptual voice quality (as judged by a certified speech-language pathologist), normal hearing screening results, normal spirometry values (> 80% predicted), no reported history of respiratory disease, no respiratory symptoms, and no smoking history within the previous 5 years.

### Statistical Analysis

Summary measures for each group are presented as means and two-sided 95% CIs for continuous (approximate) normally distributed variables and frequencies and percentages for categorical variables. For nonnormally distributed variables, bias-corrected accelerated bootstrapping was performed to produce 95% CIs for the median. Descriptive subgroup comparisons were then carried out, with CIs used to inform interpretation when possible.

This study was powered to test for a minimum difference (6 %VC) in LVI between the recruited cohort and published data for normal older adults with a two-sided two-sample *t* test. As speech breathing in presbyphonia has not yet been described, this minimum difference in LVI was selected to be more conservative than that which was identified for a different dysphonic population (teachers with vocal fatigue: ~8 %VC; Lowell et al., 2008). Applying the pooled standard deviation of LVI from previous publications (Hoit et al., 1989; Hoit & Hixon, 1987) for older adults in extemporaneous speech at conversational loudness of 8.08 %VC and assuming a significance level of 0.05, a target sample size of 35 would result in 88.6% power, balancing the feasibility of recruitment with the risk of a Type II error.

For the primary outcome, therefore, a comparison of mean LVI between the collected and published data for normophonic older adults (Hoit et al., 1989; Hoit & Hixon, 1987; Huber, 2008) was performed using two-sided, two-sample *t* tests; all model assumptions were checked. Secondary outcomes (LVT, LVE, LVE_SYL_, expiratory flow, utterance length, etc.) were compared descriptively, only. Mean differences between collected and published data, together with their 95% CIs and Cohen's *d*, are provided to aid interpretation; comparisons for which the 95% CI excludes zero are reported as evidence of a difference between the groups. However, as this study was only powered to detect differences in the primary outcome, differences reported for secondary outcomes are to be interpreted with caution.

To examine how utterance length demands affect speech breathing in people with presbyphonia, we performed an exploratory linear mixed-effects analysis using utterance-by-utterance kinematic data from the presbyphonic cohort and older adult controls (Huber, 2008). Fixed effects were cohort (presbyphonia vs. control) and condition (comfortable vs. loud); utterance length was included as a covariate. Models included random effects to account for the repeated, utterance level structure of the data. Marginal slopes are presented with their 95% CIs. Subgroup slopes (cohort × condition) are summarized using model-estimated coefficients with 95% CIs, which are used to evaluate the direction and magnitude of associations between utterance length and each outcome. As this analysis was exploratory and the study was not powered to detect subgroup or covariate effects, findings from the mixed-effects models should be interpreted with caution.

## Results

Forty-one older adults with presbyphonia were approached to take part in the study; however, five declined to take part and one was excluded due to having additional pathology (laryngeal chondroma). Thirty-five participants (20 male and 15 female participants; *M*_age_ = 79.8, range: 63–101) were consented, and all completed the study. For two participants (one man and one woman), speech breathing kinematics were so different from speechlike breathing calibration tasks (due to marked paradoxical movements) that summing of the signals would not represent a valid estimate of lung volume during speech. One female was severely dyslexic and unable to carry out the reading task. Thus, the total subjects included in the lung volume analyses are 33 for the monologue task and 32 for the reading task, somewhat smaller than the intended sample size for the analysis of our primary outcome, LVI.

### Monologue Task

Figure 2 shows mean primary and secondary speech breathing outcomes for the monologue task in comfortable and loud conditions together with their 95% CIs (full descriptive statistics are provided in Appendix A). Figure 2a shows the mean LVI for the presbyphonia cohort and the two control groups for the monologue task and demonstrates a clear pattern of low LVI for the presbyphonic participants. In the comfortable condition*,* they initiated speech at significantly lower lung volumes than both Hoit's control group (difference: −8.8 %VC; *t* = 3.0, *df* = 51, *p* = .004; *d =* −0.85) and Huber's control group (difference [diff]: −15.4 %VC; *t* = −4.6, *df* = 54, *p* < .001; *d* = −1.84). This pattern of lower LVI was also identified for the loud condition with respect to Huber's control group (diff: −11.5 %VC; *t* = −2.7, *df* = 52, *p* = .008; *d* = −0.77). When speaking against background noise in the loud condition, the presbyphonia cohort increased LVI modestly (diff: +3.12 %VC; 95% CI [0.22, 6.02], *d =* −0.38), but Huber's control group did not (diff: −0.91 %VC; 95% CI [−7.37, 5.55]; *d =* 0.06).

**Figure 2.**
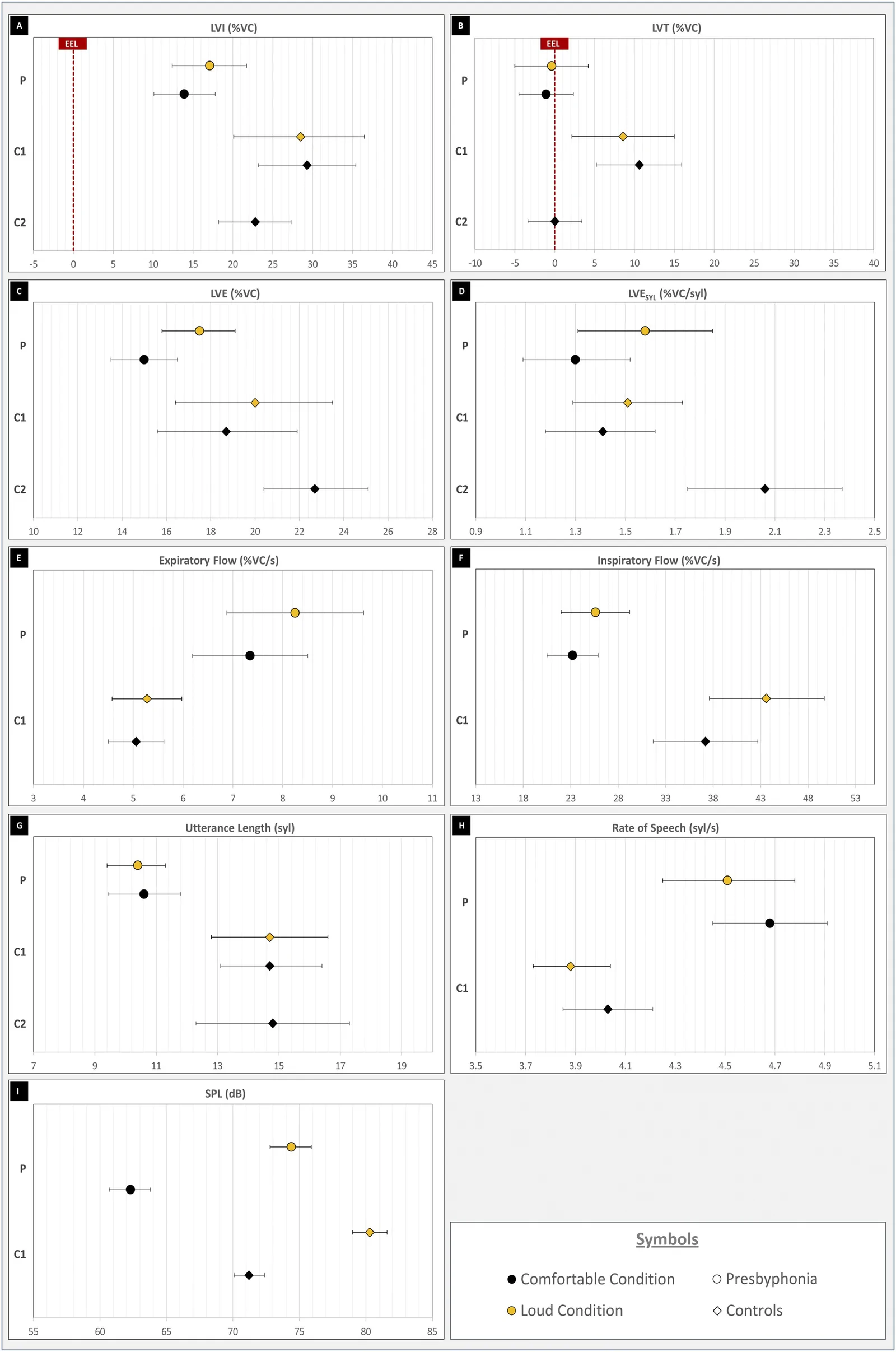
(a–i). Mean monologue speech breathing variables with 95% CIs in comfortable and loud conditions. syl = syllable; %VC = % vital capacity; P = presbyphonia; C1 = Huber; C2 = Hoit; EEL = end expiratory level; LVI = lung volume initiation; LVT = lung volume termination; LVE = lung volume excursion; LVESYL = lung volume excursion per syllable.

Figure 2b shows the mean LVT for the groups in the monologue task. In this case, the participants with presbyphonia terminated speech at lower lung volumes only with respect to Huber's control group (diff from Huber: −11.7 %VC; 95% CI [−17.6, −5.77]; *d* = −1.08), as both they and Hoit's control group terminated speech at a similar level very close to EEL (diff from Hoit: −1.10 %VC, 95% CI [−6.11, 3.92]; *d =* −0.12). This pattern was unchanged for the loud condition (diff from Huber: −8.94 %VC, 95% CI [−16.5, −1.43]; *d =* −0.67). Neither the presbyphonic cohort (diff: 0.69 %VC; 95% CI [−2.41, 3.79]; *d =* −0.08) nor Huber's control group (diff: −2.20 %VC; 95% CI [−8.65, 4.25]; *d =* 0.16) altered their LVT in the loud condition relative to the comfortable condition.

Figure 2c displays the mean LVE for the groups in the monologue task. Here, there is a trend for the presbyphonic participants to have the smallest per utterance expenditure. The difference was largest for the comfortable condition (diff from Hoit: −7.7 %VC; 95% CI [−10.3, −5.11]; *d =* −1.70; diff from Huber: −3.7 %VC; 95% CI [−6.77, −0.63]; *d =* −0.66) and somewhat smaller for the loud condition (diff: −2.53 %VC; 95% CI [−5.90, 0.84]; *d =* −0.42). Relative to the comfortable condition, the presbyphonia cohort increased LVE (diff: 2.43 %VC; 95% CI [1.56, 3.30]; *d =* 0.99) but Huber's control group did not, as CIs crossed zero (diff: 1.29 %VC; 95% CI [−0.59, 3.17]; *d =* 0.31). Despite the large effect size for this increase in LVE for presbyphonic speakers, the increase in real terms (%VC) was modest.

Figure 2d displays the mean expenditure per syllable (LVE_SYL_) for the groups. Again, the trend here was for the presbyphonic speakers to have the smallest expenditure, but true differences existed only in comparison with Hoit's control group (diff: −0.76 %VC/syl; 95% CI [−1.14, −0.38]; *d* = −1.14), as expenditure normalized per syllable was very similar to Huber's control participants in both the comfortable (diff: −0.11 %VC/syl; 95% CI [−0.41, 0.22]; *d =* −0.19) and loud (diff: −0.11 %VC/syl; 95% CI [−0.41, 0.22]; *d =* −0.19) conditions. Again, with respect to the comfortable condition, the presbyphonic speakers increased LVE_SYL_ (diff: 0.50 %VC/syl; 95% CI [0.20, 0.93]; *d =* 0.57) but Huber's control group did not (diff: 0.10 %VC/syl; 95% CI [−0.05, 0.25]; *d =* 0.31), as CIs crossed zero.

Participants with presbyphonia exhibited considerably higher expiratory flow (see Figure 2e) than Huber's control group in both loudness conditions (diff in comfortable: 2.4 %VC/s; 95% CI [0.85, 3.73]; *d =* 0.88; diff in loud: 3.0 %VC/s; 95% CI [1.20, 4.75]; *d =* 0.94). Relative to the comfortable condition, the presbyphonic cohort demonstrated increased airflow in the loud condition (diff: 0.90 %VC/s; 95% CI [0.40, 1.41]; *d =* 0.63), but controls did not (diff: 0.30 %VC/s; 95% CI [−0.21, 0.82]; *d =* −0.27).

This pattern was reversed, however, for inspiratory flow (see Figure 2f). Speakers with presbyphonia had considerably lower inspiratory flows than Huber's participants in both comfortable (diff: −14.0 %VC/s; 95% CI [−19.7, −9.08]; *d =* −1.51) and loud (diff: −18.0 %VC/s; 95% CI [−24.2, −11.4]; *d =* −1.60) conditions. As with expiratory flow, there was a trend for both groups to increase inspiratory flow in the loud condition relative to the comfortable condition, but for controls, the change was comparatively larger (diff for controls: 6.4 %VC/s; 95% CI [3.7, 9.2]; *d =* 1.07) than it was for the presbyphonia cohort (diff for presbyphonia: 2.5 %VC/s; 95% CI [0.2, 4.8]; *d =* 0.38).

Figure 2g shows mean utterance length for the groups and a clear difference between presbyphonic cohort and the controls. The presbyphonic speakers exhibited shorter utterances than control participants in the comfortable condition (diff from Hoit: −4.2 syl; 95% CI [−6.6, −1.8]; *d* = −1.00; diff from Huber: −4.1 syl; 95% CI [−6.0, −2.2]; *d* = −1.15) and the loud condition (diff: −4.3 syl; 95% CI [−6.2, −2.5]; *d* = −1.26). Utterance length was consistent for all groups regardless of loudness condition, as neither the presbyphonic speakers (mean diff: −0.24 syl; 95% CI [−1.13, 0.65]; *d =* 0.09) nor Huber's control group (mean diff: −0.26 syl; 95% CI [−1.40, 0.87]; *d =* 0.10) demonstrated any change when speaking in background noise.

Mean rate of speech is found in Figure 2h, which demonstrates that participants with presbyphonia spoke ~15% faster than Huber's control group in both in the comfortable (diff: 0.58 syll/s; 95% CI [0.34, 0.96]; *d =* 1.06) and loud (diff: 0.63 syl/s; 95% CI [0.28, 0.99]; *d =* 1.00) conditions. There was a trend for both groups to slow their speech in the loud condition relative to the comfortable condition (diff for presbyphonia: −0.17 syl/s; 95% CI [−0.32, −0.01]; *d = −*0.36; diff for controls: −0.17 syl/s; 95% CI [−0.33, −0.02]; *d = −*0.50), but this was perhaps modest in magnitude.

Finally, Figure 2i shows mean SPL for the presbyphonic and Huber control cohorts. Here, there is a clear trend for the speakers with presbyphonia to be considerably quieter than controls in both loudness conditions (diff in comfortable: −8.9 dB; 95% CI [−11, −6.9]; *d =* −2.27; diff in loud: −5.9 dB; 95% CI [−8.1, −3.7]; *d* = −1.49), although the disparity narrowed in the loud condition. Relative to the comfortable condition, both groups increased speech intensity by a considerable margin when speaking in background noise (diff in presbyphonia: 12 dB; 95% CI [11, 13]; *d =* 3.51; diff in Huber: 8.9 dB; 95% CI [7.9, 10]; *d =* 3.91).

### Reading Task

Figure 3 shows mean speech breathing outcomes during reading together with their 95% CIs, compared to the Hoit data sets, who also used the *California Passage*, offering a direct comparison (descriptive statistics are provided in Appendix B). Comparison of speech breathing outcomes between loudness conditions is restricted to the cohort with presbyphonia, as the Hoit data sets did not include loud oral reading.

**Figure 3.**
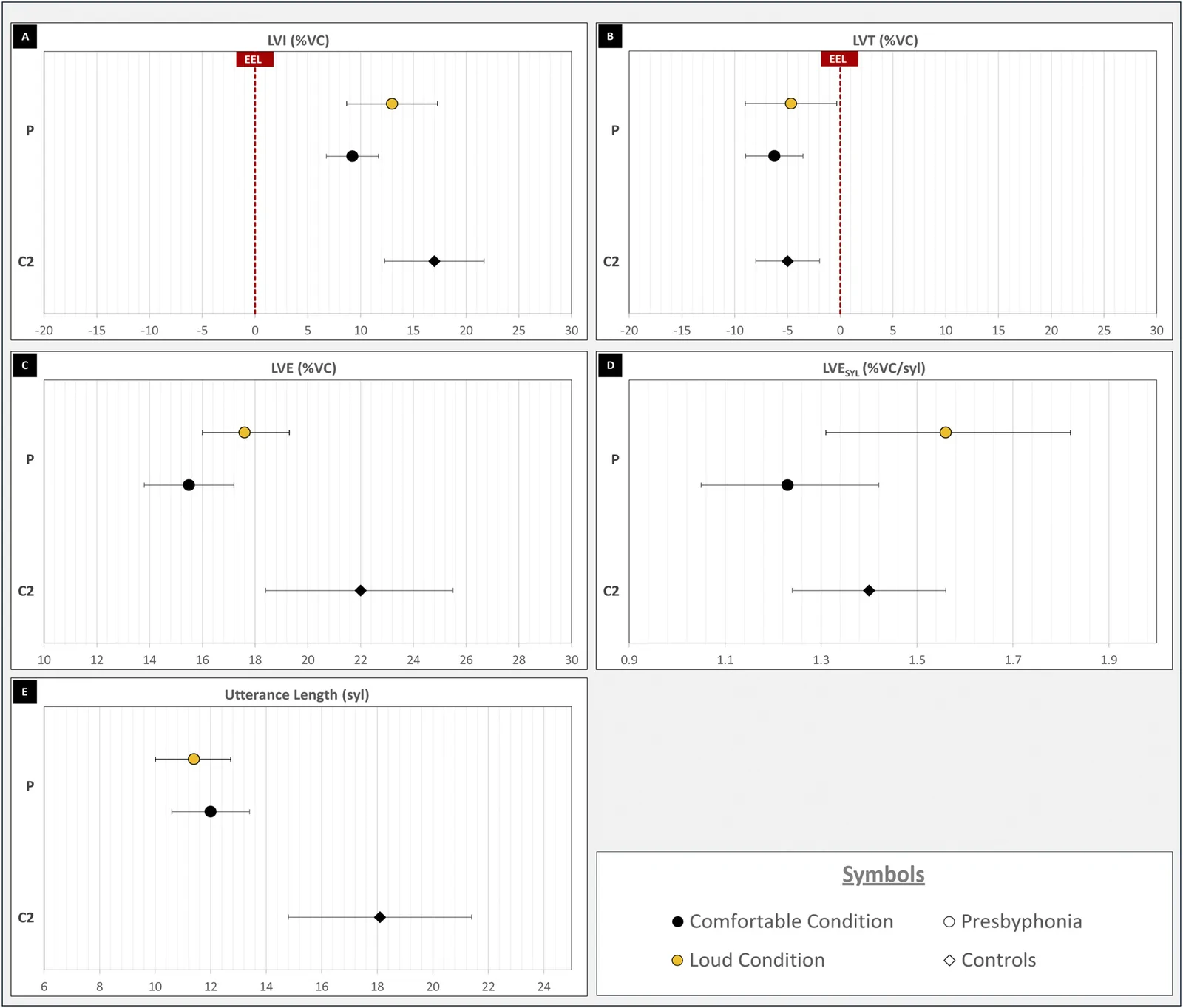
(a–e). Mean reading speech breathing variables with 95% CIs in comfortable and loud conditions. syl = syllable; %VC = % vital capacity; P = presbyphonia; C2 = Hoit; EEL = end expiratory level; LVI = lung volume initiation; LVT = lung volume termination; LVE = lung volume excursion; LVESYL = lung volume excursion per syllable.

Figure 3a shows mean LVI for oral reading. As with the monologue task, participants with presbyphonia initiated oral reading significantly lower than controls (diff: −7.8% VC; *t* = 3.6, *df =* 51, *p* < .001; *d =* −1.01). Also like the monologue data, presbyphonic participants modestly increased LVI in the loud condition (diff: 3.76 %VC; 95% CI [0.44, 7.08]; *d =* 0.41).

Figure 3b indicates that for the reading task, there was no apparent difference in LVT between presbyphonic speakers and controls as both terminated speech at ~5 %VC below EEL (diff from controls: −1.25 %VC; 95% CI [−5.32, 2.82]; *d =* −0.17). There was no change in LVT for presbyphonic speakers in the loud condition (diff: 1.58 %VC; 95% CI [−1.62, 4.78]; *d =* −0.18) as CIs crossed zero.

For LVE (see Figure 3c), speakers with presbyphonia again utilized much less of their VC per utterance than controls in oral reading (diff: −6.50 %VC; 95% CI [−9.89, −3.11]; *d =* −1.10). As with the monologue task, they demonstrated a modest increase in LVE in the loud condition relative to the comfortable condition (diff: 2.18 %VC; 95% CI [1.07, 3.30]; *d =* 0.71).

When normalized per syllable (LVE_SYL_), however, this difference in expenditure from controls did not persist as is evident by the overlapping CIs in Figure 3d. Participants with presbyphonia expended a similar VC per syllable (diff from controls: −0.17 %VC/syl; 95% CI [−0.43, 0.09]; *d =* −0.37). Once again as found for the monologue task, presbyphonic speakers increased LVE_SYL_ in the loud condition relative to the comfortable condition (diff: 0.33 %VC/syl; 95% CI [0.22, 0.44]; *d =* 1.06).

Mean utterance length data (see Figure 3e) demonstrated that even when reading aloud an identical stimulus passage, participants with presbyphonia again produced considerably shorter utterances than controls (diff: −6.1 syl; 95% CI [−9.1, −3.1]; *d =* −1.15). Utterance length also did not change in the loud relative to the comfortable condition (diff: −0.62 syl; 95% CI [−1.48, 0.25]; *d =* 0.25).

### Influence of Utterance Length on Monologue Speech

Figure 4 presents the marginal subgroup slopes of the exploratory mixed-effects analysis of utterance length on speech breathing outcomes in the monologue task for the presbyphonia cohort and for Huber's control group. Descriptive statistics for slopes and a full table of model coefficients are available in Appendix C.

**Figure 4.**
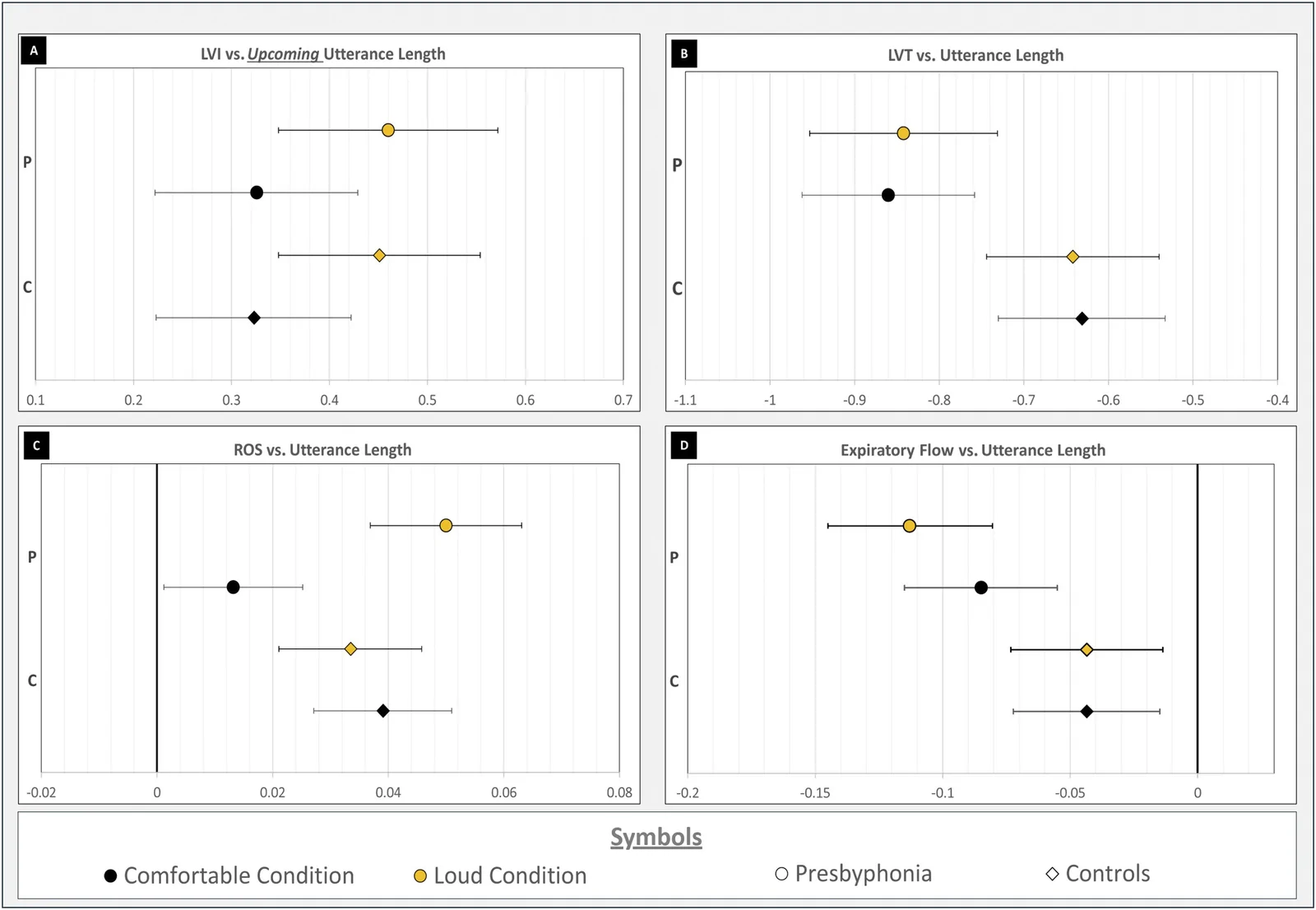
(a–d). Marginal slopes and 95% CIs for mixed-effects models of the influence of utterance length on speech breathing. P = presbyphonia; C = controls; LVI = lung volume initiation; LVT = lung volume termination; ROS = rate of speech.

Figure 4a shows the relationship that upcoming utterance length had on LVI for the two groups in the two conditions, revealing a remarkably similar picture for presbyphonic speakers and controls. Utterance length showed a reliable positive association with LVI (β = .32, 95% CI [.22, .42]), indicating that LVI generally increased with increasing upcoming utterance length. The considerable overlap of CIs, however, indicates that the strength of this relationship did not differ between cohorts (presbyphonia vs. control) or loudness conditions (comfortable vs. loud). Specifically, the difference in slopes between cohorts in the comfortable condition was near zero (Δβ = .003, 95% CI [−.14, .15]), and the impact of utterance length on LVI by cohort did not change in the loud condition, as reflected by a nonsignificant three-way interaction between cohort, condition, and utterance length (β = .006, 95% CI [−.20, .21]).

Figure 4b shows the relationship between LVT and the length of utterance, which was negative for all participants (β = −.63, 95% CI [−.73, −.53]), indicating that LVT generally decreased as utterance length increased, but the relationship was stronger for presbyphonic participants (Δβ = −.23, 95% CI [−.37, −.09]). As evident in the complete overlap of CIs within the two cohorts, the impact of utterance length on LVT did not vary by loudness condition, as indicated by a nonsignificant Cohort × Condition × Utterance Length interaction (β = .03, 95% CI [−.17, .23]).

Figure 4c shows the relationship between speech rate and utterance length, which was positive for all participants (β = .039, 95% CI [.027, .051]), indicating that longer upcoming utterances were generally associated with faster speech rates. The pattern of CIs in Figure 4c, however, shows a complex relationship between cohort and condition for the strength of this association. In the comfortable condition, participants with presbyphonia exhibited a shallower increase in rate of speech with increasing utterance length compared to controls (Δβ = −.026, 95% CI [−.043, −.009]), but a steeper increase in the loud condition. This differential impact is reflected in a positive Cohort × Condition × Utterance Length interaction (β = .042, 95% CI [.019, .066]).

Finally, Figure 4d shows the relationship between expiratory flow and utterance length, which was negative for all participants (β = −.043, 95% CI [−.072, −.015]), indicating that flow tended to decrease as utterance length increased. The overall trend evident in the data was for the relationship to be more negative for presbyphonic speakers, and this was borne out in linear mixed-effects modeling (Δβ = −.041, 95% CI [−.083, .000]), although the CI narrowly included zero. Even though the most negative relationship was found for the presbyphonia cohort in the loud condition, a nonsignificant Cohort × Condition × Utterance Length interaction (β = −.028, 95% CI [−.086, .030]) indicates there was not a significant differential impact of loudness on the association.

## Discussion

This study provides the first breath-by-breath characterization of speech breathing kinematics in older adults with presbyphonia. Across tasks and loudness conditions, speakers initiated phonation at substantially lower lung volumes than age-matched, vocally healthy adults and produced smaller LVEs. They also generated shorter yet faster utterances with elevated expiratory airflow and markedly slower inspiratory flows. These features were consistent across reading and monologue tasks and partially normalized in background noise, indicating responsiveness to increased loudness demands. At the utterance level, participants adjusted LVI, LVT, expiratory flow, and rate of speech in relation to utterance length suggesting intact anticipatory planning and online adaptation. Collectively, the findings suggest the possibility of a distinct respiratory profile in presbyphonia, characterized by reduced LVI, constrained LVE, and compensatory temporal and airflow strategies.

LVI, the primary outcome, was 9%–15% VC lower in presbyphonic speakers than in normative cohorts, aligning them with other dysphonic populations who demonstrate reduced inspiratory initiation: teachers with voice disorders (Lowell et al., 2008), women with vocal abuse-related dysphonia (Schaeffer et al., 2002), speakers with high vocal fatigue (Fujiki et al., 2022), and adults with later stage Parkinson's disease (Huber & Darling-White, 2017). Unlike many such groups, however, participants did not reduce LVT to maintain excursion; instead, they produced consistently smaller LVEs. The combination of low LVI and reduced LVE therefore constrained both the usable volume and the passive recoil pressures available for speech.

Despite lower LVIs on average, anticipatory control was intact as the presbyphonic group increased LVI when longer upcoming utterances were planned, indicating preserved planning mechanisms (Huber & Spruill, 2008; Sperry & Klich, 1992; Winkworth et al., 1994, 1995). They also demonstrated a similar, if somewhat more pronounced, lowering of LVT with increasing utterance length, indicating online and adaptive flexibility in responding to utterance length demands (Huber, 2008; Huber & Darling, 2011; Schaeffer et al., 2002; Winkworth et al., 1994, 1995). Presbyphonic speakers, therefore, did not demonstrate a loss of anticipatory speech breathing control or flexibility, instead they adapted their speech breathing to a narrower physiological operating range.

One adaptive strategy was the temporal restructuring of phrasing, involving both the shortening and speeding up of utterances. Across tasks and conditions, presbyphonic speakers produced consistently shorter utterances. Shorter utterances persisted even during reading, suggesting that temporal restructuring reflects physiological necessity rather than linguistic planning alone. Additionally, longer utterances were produced consistently and across conditions with a faster rate of speech by both healthy and presbyphonic participants. In short, it seems that in a constrained environment of smaller LVEs and higher expiratory airflow, speakers with presbyphonia increased speed and tightened up phrasing to maximize communicative throughput given the resources available. Such compensation confirms that the role of linguistic planning in speech breathing is not just one way, whereby lung volumes are planned in anticipation of utterance length. As identified by Włodarczak and Heldner (2017), linguistic planning (in this case adapting speech rate to utterance length) is also impacted by the “momentary respiratory state” of the system, that is, the volume available at the moment of speaking.

Flow modulation was another adaptive strategy. Longer utterances were associated with reduced expiratory flow in both groups, but the decline was more pronounced in presbyphonic speakers, implying perhaps a greater need to conserve air by restricting flow. Presbylarynx is frequently associated with glottic incompetence and a spindle-shaped glottis (Kost & Sataloff, 2020), which finds support in our cohort's high expiratory flow compared to controls. However, despite the presence of persistent glottal gap in 47% of our participants, nearly all of them (97%) demonstrated compensatory hyperfunction, a common finding in vocal fold atrophy (Belafsky et al., 2002). Unlike other populations with glottal incompetence that compensate for high flow by increasing LVE (Heller Murray et al., 2017; Sapienza & Stathopoulos, 1994), speakers with presbyphonia did not. Increasing LVE by increasing LVIs may have been disadvantageous for flow regulation in light of glottic incompetence as higher lung volumes are associated with increased tracheal pull that widens the glottis and reduces vocal fold contact time and airway resistance (Baier et al., 1977; Iwarsson et al., 1998; Stănescu et al., 1972). Increasing LVE by lowering LVTs would also have been disadvantageous as LVTs were already close to EEL, and terminating below this level is rarely observed in older adults (Hoit et al., 1989; Hoit & Hixon, 1987; Huber & Spruill, 2008; Sperry & Klich, 1992; Sundarrajan et al., 2017; Winkworth et al., 1994, 1995). This is because such lower terminations would be progressively more fatiguing once EEL is crossed and recoil pressures become negative, which would especially be the case considering our participants' elevated subglottic pressure relative to norms. As such, maintaining a smaller LVE may have been an adaptive flow modulation compromise between reduced valving on the one hand and increased effort on the other.

There are several other potential explanations for why speakers with presbyphonia might not opt for a strategy of higher LVIs like their normophonic controls. Speech breathing patterns can reflect learned or habituated motor behaviors in the absence of a physiological cause; maladaptive patterns similar to those reported here have been described in pMTD (Lowell et al., 2008, 2025). On the other hand, inspiratory flows were consistently reduced: Participants inhaled both more slowly (diff: 0.16 s; 95% CI [0.07, 0.25]; *d =* 0.98) and to smaller volumes than controls (diff: −4.9 %VC; 95% CI [−8.6, −1.2]; *d =* −0.73). This pattern of slow inhalations is the same as identified by Loudon et al. (1988) in adults with asthma, hypothesized to be the result of giving gas exchange priority over speech production. As 20% of our cohort presented with obstructive spirometry, it is possible that airflows were similarly restricted for this reason or other disease-specific reasons (e.g., COPD can lead to hyperinflation, making rapid inspiratory flows harder to achieve [O'Donnell & Laveneziana, 2006], and interstitial lung disease increases the work of inspiration and limits inspiratory flow velocity; Plantier et al., 2018). There is also the potential mechanical impact of age-related thoracic and postural changes that alter the geometry and compliance of the chest wall, thereby making higher lung volumes more challenging to achieve (Mandros et al., 2008).

These potential physiological mechanisms also raise a methodological consideration: The respiratory profile of our cohort may itself have influenced these patterns. Although including older adults with respiratory comorbidities enhances ecological validity, it also introduces a potential limitation as comparison cohorts excluded individuals with respiratory disease. In our sample, the prevalence of respiratory conditions and smoking history were roughly twice that of U.K. national estimates (Office for National Statistics, 2023; Whittaker et al., 2025), and one fifth of participants demonstrated abnormal spirometry.

However, exploratory analyses actually suggested the opposite of what might be expected if respiratory status were confounding LVI behavior. Across the cohort, better respiratory function was associated with more age-atypical LVI patterns. Raw measures of FEV_1_, FVC, and MIP were negatively correlated with LVI (FEV_1_: *r* = −.495, *p* = .003; FVC: *r* = −.488, *p* = .004; MIP: *r* = −.398, *p* = .02), with a similar pattern for standardized indices (FEV_1_ *z* score: *r* = −.316, *p* = .07; FVC *z* score: *r* = −.391, *p* = .03; % predicted MIP: *r* = −.311, *p* = .08). Inspiratory flow showed the same direction of effect: Higher (more normative) flow was associated with poorer spirometry (FEV_1_: *r* = −.398, *p* = .02; FVC: *r* = −.400, *p* = .02; FEV_1_ *z* score: *r* = −.515, *p* = .002; FVC *z* score: *r* = −.619, *p* < .001). Taken together, these findings indicate that participants with larger or healthier vital capacities, less airway obstruction, and stronger inspiratory musculature were more likely to exhibit low LVI and reduced inspiratory flows. Conversely, those with more constrained respiratory capacity tended to initiate speech higher in their VC range, potentially because reduced physiological reserve required them to operate closer to their limits during speech tasks.

If presbyphonia reflects an adaptive reorganization of speech breathing, rather than a purely laryngeal deficit, then interventions that target respiratory support and breathing behavior may meaningfully complement traditional laryngeal approaches. Emerging evidence already points in this direction. Respiratory lung volume training, which uses biofeedback to increase LVI, has produced promising outcomes in pMTD (Lowell et al., 2022, 2025). Similarly, incorporating inspiratory muscle strength training into presbyphonia therapy has yielded greater improvements in voice quality, vocal effort, and patient-reported outcomes than treatments without such training (Desjardins et al., 2022b; Yao et al., 2025). Although neither study measured speech kinematics, both sets of authors propose that strengthening inspiratory behavior may facilitate higher LVIs, greater passive recoil forces, and reduced reliance on laryngeal valving. Additionally, as our participants demonstrated more normative LVI, LVE, inspiratory flow, LVE_SYL_, and slower speech rates when speaking in background noise (conditions that naturally elicit increased loudness), interventions for presbyphonia incorporating increased loudness such as Phonation Resistance Training Exercises (Ziegler et al., 2014) may also align with the underlying respiratory–laryngeal dynamics of presbyphonia.

This study has several strengths: It is the first breath-by-breath characterization of speech breathing in older adults with clinician-confirmed presbyphonia, using carefully calibrated inductive plethysmography together with concurrent acoustic capture and validated respiratory calibration procedures. However, several limitations should also be borne in mind. Comparisons relied on historical and published control data collected at different times and, in the case of the Hoit data sets, with some subtly different instrumentation (magnetometer vs. respiratory inductive plethysmography) and calibration methods (iso-volumes vs. least squares). Despite these data enabling an analysis that would have otherwise only been possible with the increased burden of a prospective control sample, the use of historical data sets may have introduced unavoidable cohort differences. Additionally, our sample was modest in size and ethnically homogenous, which limits both statistical power outside our primary outcome and generalizability to diverse populations. Finally, despite normalizing SPL values to the same virtual 30-cm distance, small differences in microphone placement may have affected intensity-level comparisons. Furthermore, even though both normophonic and presbyphonic participants were speaking English, the differing sociolinguistic contexts (the United States vs. the United Kingdom) means that culturally mediated social norms may have been a confounder in observed differences in vocal intensity.

## Conclusions

Our sample of older adults with presbyphonia showed a consistent pattern of altered speech breathing compared to controls: They initiated phonation at lower lung volumes, expended less volume per utterance, produced shorter but faster utterances, and displayed slowed inhalation alongside relatively elevated expiratory airflow. These patterns persisted across reading and spontaneous speech and partially normalized under louder speaking conditions. These findings indicate that disturbed respiratory kinematics may contribute to the clinical picture of presbyphonia. Clinically, assessment and rehabilitation of presbyphonia should explicitly include kinematic evaluation and consider speech breathing alongside laryngeal factors. Future work should recruit contemporaneous, well-characterized controls, objectively quantify respiratory disease severity, and test whether respiratory-focused therapies improve both breathing kinematics and voice outcomes.

## Author Contributions

**Brian Saccente-Kennedy:** Conceptualization, Methodology, Investigation, Data curation, Project administration, Funding acquisition, Software, Visualization, Writing – original draft, Writing – review & editing. **Jessica E. Huber:** Methodology, Supervision, Data curation, Writing – review & editing. **Maude Desjardins:** Writing – review & editing. **Martin Wiegand:** Formal analysis, Visualization, Writing – review & editing. **Matthew Maddocks:** Supervision, Writing – review & editing. **Roganie Govender:** Supervision, Writing – review & editing.

## Data Availability Statement

The data that support the findings of this study are available from the corresponding author upon reasonable request.

## Appendix A

### Monologue Task

**Table:**

*M* ± *SD* [95% CI]	Comfortable			Loud	
	Presbyphonia (*N* = 35)	Huber data set (*n* = 23)	Hoit data sets (*n* = 20)	Presbyphonia (*N* = 35)	Huber data set (*n* = 23)
LVI (%VC)	13.9 ± 10.9 [10.1, 17.8]	29.3 ± 14.0 [23.2, 35.4]	22.8 ± 9.67 [18.2, 27.3]	17.1 ± 13.1 [12.4, 21.7]	28.5 ± 17.6 [20.1, 36.5]
LVT (%VC)	−1.08 ± 9.63 [−4.49, 2.33]	10.6 ± 12.4 [5.22, 15.9]	0.015 ± 7.22 [−3.36, 3.39]	−0.393 ± 13.0 [−5.00, 4.21]	8.55 ± 14.1 [2.14, 15.0]
LVE (%VC)	15.0 ± 4.21 [13.5, 16.5]	18.7 ± 7.24 [15.6, 21.9]	22.7 ± 5.07 [20.4, 25.1]	17.5 ± 4.57 [15.8, 19.1]	20.0 ± 7.79 [16.4, 23.5]
LVE_SYL_ (%VC/syl)	1.30 ± 0.64 [1.09, 1.52]	1.41 ± 0.48 [1.18, 1.62]	2.06 ± 0.71 [1.75, 2.37]	1.58 ± 0.78 [1.31, 1.85]	1.51 ± 0.48 [1.29, 1.73]
Expiratory flow (%VC/s)	7.34 ± 3.26 [6.19, 8.50]	4.97 ± 1.29 [4.39, 5.55]	Not reported	8.25 ± 3.86 [6.88, 9.62]	5.28 ± 1.54 [4.58, 5.98]
Inspiratory flow (%VC/s)	23.2 ± 0.07 [20.5, 25.9]	37.2 ± 0.12 [31.7, 42.7]	Not reported	25.6 ± 0.10 [22.0, 29.2]	43.6 ± 0.13 [37.6, 49.7]
Utterance length (syl)	10.6 ± 3.41 [9.43, 11.8]	14.7 ± 3.82 [13.1, 16.4]	14.8 ± 5.29 [12.3, 17.3]	10.4 ± 2.78 [9.40, 11.3]	14.7 ± 4.21 [12.8, 16.6]
Rate of speech (syllables/s)	4.68 ± 0.67 [4.45, 4.91]	4.03 ± 0.41 [3.85, 4.21]	Not reported	4.51 ± 0.76 [4.25, 4.78]	3.88 ± 0.34 [3.73, 4.04]
Intensity (dB SPL_30cm_)	62.3 ± 4.56 [60.7, 63.8]	71.2 ± 2.66 [70.1, 72.4]	Not reported	74.4 ± 4.51 [72.8, 75.9]	80.3 ± 2.94 [79.0, 81.6]

*Note.* CI = confidence interval; LVI = lung volume initiation; %VC = % vital capacity; LVT = lung volume termination; LVE = lung volume excursion; LVE_SYL_ = LVE per syllable; syl = syllable.

## Appendix B

### Reading Task

**Table:**

*M* ± *SD* [95% CI]	Comfortable		Loud
	Presbyphonia (*n* = 35)	Hoit data sets (*n* = 20)	Presbyphonia (*N* = 35)
LVI (%VC)	9.22 ± 6.81 [6.77, 11.7]	17.0 ± 8.97 [12.3, 21.7]	13.0 ± 11.9 [8.70, 17.3]
LVT (%VC)	−6.24 ± 7.53 [−8.96, −3.53]	−4.99 ± 6.46 [−8.01, −1.96]	−4.66 ± 12.0 [−9.00, −0.326]
LVE (%VC)	15.5 ± 4.72 [13.8, 17.2]	22.0 ± 7.59 [18.4, 25.5]	17.6 ± 4.60 [16.0, 19.3]
LVE_SYL_ (%VC/syl)	1.23 ± 0.51 [1.05, 1.42]	1.40 ± 0.37 [1.24, 1.56]	1.56 ± 0.71 [1.31, 1.82]
Utterance length (syl)	12.0 ± 3.99 [10.6, 13.4]	18.1 ± 7.10 [14.8, 21.4]	11.4 ± 3.88 [7.99, 12.1]

*Note.* CI = confidence interval; LVI = lung volume initiation; %VC = % vital capacity; LVT = lung volume termination; LVE = lung volume excursion; LVE_SYL_ = LVE per syllable; syl = syllable.

## Appendix C

### Mixed-Effects Models

**Table C1.** Marginal subgroup slopes for mixed-effects analysis of utterance length (P = presbyphonia; C = controls).

		Comfortable			Loud		
		Slope	*SE*	95% CI	Slope	*SE*	95% CI
**LVI vs. upcoming length**	P	0.326	0.053	[0.222, 0.429]	0.460	0.057	[0.348, 0.572]
(%VC × syl)	C	0.323	0.051	[0.223, 0.422]	0.451	0.053	[0.348, 0.554]
**LVT vs. length**	P	−0.860	0.052	[−0.962, −0.758]	−0.842	0.057	[−0.953, −0.731]
(%VC × syl)	C	−0.631	0.050	[−0.730, −0.533]	−0.642	0.052	[−0.744, −0.540]
**Rate of speech vs. length**	P	0.013	0.006	[0.001, 0.025]	0.050	0.007	[0.037, 0.063]
(syl/s × syl)	C	0.039	0.006	[0.027, 0.051]	0.034	0.006	[0.021, 0.046]
**Expiratory flow vs. length**	P	−0.085	0.015	[−0.115, −0.055]	−0.113	0. 017	[−0.145, −0.081]
(%VC/s × syl)	C	−0.044	0.015	[−0.072, −0.003]	−0.044	0.015	[−0.073, −0.014]

*Note.* CI = confidence interval; LVI = lung volume initiation; %VC = % vital capacity; syl = syllable; LVT = lung volume termination.

**Table C2.** Full table of mixed-effect models coefficients.

Term	Est.	95% CI-L	95% CI-H	*p* value	*SE*	*t*	*df*
LVI vs. upcoming utterance length							
(Intercept)	24.8340	19.2994	30.3685	.0000	2.7706	8.9634	64.2337
Cohort(P)	−14.2697	−21.4507	−7.0887	.0002	3.5936	−3.9708	63.1362
Cond(L)	−3.8430	−6.1783	−1.5077	.0013	1.1911	−3.2265	3373.4271
Length	0.3225	0.2228	0.4222	.0000	0.0508	6.3436	3377.3631
Cohort(P) × Cond (L)	5.5570	2.6705	8.4436	.0002	1.4722	3.7746	3371.9486
Cohort(P) × Length	0.0030	−0.1405	0.1466	.9671	0.0732	0.0412	3376.7265
Cond(L) × Length	0.1288	−0.0091	0.2667	.0671	0.0703	1.8319	3369.0414
Cohort(P) × Cond(L) × Length	0.0055	−0.1955	0.2064	.9575	0.1025	0.0532	3369.0886
LVT vs. utterance length							
(Intercept)	20.2175	15.4680	24.9671	.0000	2.3804	8.4934	68.3308
Cohort(P)	−12.3203	−18.4746	−6.1661	.0002	3.0831	−3.9961	66.7838
Cond(L)	−3.4714	−5.7849	−1.1578	.0033	1.1800	−2.9419	3375.7348
Length	−0.6314	−0.7302	−0.5327	.0000	0.0504	−12.5373	3380.9514
Cohort(P) × Cond (L)	3.6254	0.7656	6.4852	.0130	1.4586	2.4856	3373.7674
Cohort(P) × Length	−0.2285	−0.3707	−0.0863	.0016	0.0725	−3.1508	3380.1359
Cond(L) × Length	−0.0103	−0.1469	0.1264	.8829	0.0697	−0.1473	3369.8584
Cohort(P) × Cond(L) × Length	0.0283	−0.1709	0.2274	.7806	0.1016	0.2785	3369.9274
Rate of speech vs. utterance length							
(Intercept)	3.4359	3.1638	3.7080	.0000	0.1380	24.9006	200.1883
Cohort(P)	1.3179	0.9759	1.6600	.0000	0.1734	7.6025	182.6244
Cond(L)	−0.0580	−0.3389	0.2229	.6857	0.1433	−0.4047	3526.4066
Length	0.0391	0.0271	0.0510	.0000	0.0061	6.4071	3536.9504
Cohort(P) × Cond (L)	−0.6943	−1.0388	−0.3498	.0001	0.1757	−3.9519	3519.7454
Cohort(P) × Length	−0.0259	−0.0428	−0.0090	.0027	0.0086	−2.9983	3536.7617
Cond(L) × Length	−0.0056	−0.0222	0.0111	.5106	0.0085	−0.6580	3500.1344
Cohort(P) × Cond(L) × Length	0.0424	0.0185	0.0663	.0005	0.0122	3.4829	3500.8554
Expiratory flow vs. utterance length							
(Intercept)	5.9220	4.5765	7.2675	.0000	0.6746	8.7787	69.7475
Cohort(P)	2.7171	0.9742	4.4600	.0027	0.8734	3.1109	68.0765
Cond(L)	0.3726	−0.3022	1.0474	.2790	0.3442	1.0827	3376.5413
Length	−0.0435	−0.0723	−0.0147	.0031	0.0147	−2.9647	3382.0306
Cohort(P) × Cond (L)	0.7908	−0.0433	1.6249	.0631	0.4254	1.8588	3374.4687
Cohort(P) × Length	−0.0414	−0.0828	0.0001	.0507	0.0212	−1.9549	3381.1798
Cond(L) × Length	0.0000	−0.0398	0.0399	.9987	0.0203	0.0016	3370.3401
Cohort(P) × Cond(L) × Length	−0.0281	−0.0862	0.0300	.3433	0.0296	−0.9479	3370.4144

*Note.* Est. = estimated regression coefficient; CI-L = lower bound of confidence interval, CI-H = upper bound of confidence interval; LVI = lung volume initiation; P = presbyphonia; L = loud; Cond = condition; LVT = lung volume termination.
